# Estrogen Promotes Mandibular Condylar Fibrocartilage Chondrogenesis and Inhibits Degeneration via Estrogen Receptor Alpha in Female Mice

**DOI:** 10.1038/s41598-018-26937-w

**Published:** 2018-06-04

**Authors:** Jennifer L. Robinson, Paola Soria, Manshan Xu, Mark Vrana, Jeffrey Luchetti, Helen H. Lu, Jing Chen, Sunil Wadhwa

**Affiliations:** 1 Columbia University College of Dental Medicine, 622 West 168th Street, New York, NY 10032 USA; 2 Columbia University Division of Orthodontics, 622 West 168th Street, New York, NY 10032 USA; 3 Columbia University Department of Biomedical Engineering, 351 Engineering Terrace, 1210 Amsterdam Avenue, New York, NY 10027 USA

## Abstract

Temporomandibular joint degenerative disease (TMJ-DD) is a chronic form of TMJ disorder that specifically afflicts people over the age of 40 and targets women at a higher rate than men. Prevalence of TMJ-DD in this population suggests that estrogen loss plays a role in the disease pathogenesis. Thus, the goal of the present study was to determine the role of estrogen on chondrogenesis and homeostasis via estrogen receptor alpha (ERα) during growth and maturity of the joint. Young and mature WT and ERαKO female mice were subjected to ovariectomy procedures and then given placebo or estradiol treatment. The effect of estrogen via ERα on fibrocartilage morphology, matrix production, and protease activity was assessed. In the young mice, estrogen via ERα promoted mandibular condylar fibrocartilage chondrogenesis partly by inhibiting the canonical Wnt signaling pathway through upregulation of sclerostin (Sost). In the mature mice, protease activity was partly inhibited with estrogen treatment via the upregulation and activity of protease inhibitor 15 (Pi15) and alpha-2-macroglobulin (A2m). The results from this work provide a mechanistic understanding of estradiol on TMJ growth and homeostasis and can be utilized for development of therapeutic targets to promote regeneration and inhibit degeneration of the mandibular condylar fibrocartilage.

## Introduction

Temporomandibular joint degenerative disease (TMJ-DD) is marked by degradation and premature calcification of the extracellular matrix (ECM) of the articular mandibular condylar fibrocartilage. Patients with TMJ-DD experience pain during jaw movement (e.g. mastication and speaking) and are at higher risk for complete degradation of the joint and replacement surgery. TMJ-DD is a chronic form of TMJ disorder that specifically afflicts older patients and targets women at a higher rate than men. Specifically, 70% of people with TMJ-DD are 40–70 years old^1^ with females 2–3 times more likely to suffer^2^. These alarming statistics suggest that the loss of estrogen during menopause may potentiate TMJ-DD. However, little is known regarding the role of estrogen in mediating TMJ growth, homeostasis, and degeneration.

The mandibular condylar fibrocartilage functions as a growth plate cartilage to allow for longitudinal growth of the condyle and transitions to an articular cartilage after skeletal maturation^3^. The articular fibrocartilage is comprised of fibrochondrocytes that produce collagen type 1 and 2 (Col1 and Col2) and proteoglycans. Degenerating joints are marked by degradation of this collagenous and proteoglycan-rich fibrocartilage matrix. Thus, it is crucial to investigate estrogen’s signaling effects on the synthesis and maintenance of the articular fibrocartilage extracellular matrix in both skeletally immature and mature tissue to delineate its role throughout aging and determine potential therapeutic targets.

Estrogen modulates transcription via both classical and nonclassical pathways. In the classical pathway, estrogen binds to estrogen receptor alpha (ERα) or beta (ERβ) which results in a conformational change of the receptors, receptor dimerization, and translocation into the nucleus^4^. The receptor complex then typically binds to the estrogen response element (ERE) and acts as an enhancer, recruiting cofactors to promote gene transcription^5,6^. In the nonclassical pathway, ERs, either dependently or independently of ligand binding, interact with other transcriptional pathways through protein-protein interactions likely involving phosphorylation modifications^7,8^. In other musculoskeletal tissues such as bone and hyaline cartilage, ERα is required for estrogen’s anabolic effects during development and remodeling to maintain homeostasis^9–11^. On the other hand, ERβ acts as a dominant negative regulator that can replace some of ERα’s roles in its absence^12–14^. However, the role of ERα on the growth and remodeling of the mandibular condylar fibrocartilage in adults is unclear. ERα is expressed in all cells of the mandibular condylar fibrocartilage emphasizing the importance this receptor must play in estrogen signaling in the TMJ^15^. As such, it is necessary to determine the role estrogen via ERα plays on growing and mature TMJ tissue.

Most of the studies investigating estrogen’s effects on the TMJ have been conducted in young rodents^16–19^. Results from these studies show cells of the mandibular condylar fibrocartilage respond to estradiol treatment resulting in a decrease in fibrocartilage cell proliferation and an increase in chondrogenesis^17,19^. Previous studies indicated that ERβ mediated estrogen’s role on condylar fibrocartilage cell proliferation but not the chondrogenic matrix effects suggesting the role of ERα in estrogen-mediated chondrogenesis^19^. Further, recent studies from our laboratory illustrated ERα regulates mandibular condylar fibrocartilage maturation in young male mice but does significantly play a role in mediating growth or remodeling in old male mice^20^. To our knowledge, the only study conducted that investigates the effect of estrogen on skeletally mature, female rodents was completed by Talwar *et al*.^21^. In this study, estradiol treatment in *ex vivo* and *in vitro* experiments revealed a decrease in fibrocartilage thickness and cell proliferation similar to effects seen in the growing mandibular condylar fibrocartilage. However, to date, there have not been any *in vivo* studies investigating the role of ERα in skeletally mature female mice. These results warrant the need to understand estrogen’s role via ERα on homeostasis and remodeling of the mandibular condylar fibrocartilage in adult female mice.

Thus, the goal of the present study was to determine the role of estrogen on mandibular condylar chondrogenesis and homeostasis via ERα during growth and maturity of the joint. Young and mature WT and ERαKO female mice were subjected to ovariectomy procedures and then given placebo or estradiol treatment. An ovariectomy model was employed to specifically evaluate the role of estrogen through ERα on the female condylar fibrocartilage. This is vital as ERαKO mice have elevated estrogen levels due to the negative feedback loop. Thus, in this model, endogenous estrogen is no longer produced and controlled concentrations of estradiol are given to the mice. The effect of estrogen via ERα on cartilage morphology, cell proliferation, cartilaginous matrix production, protease activity, and extracellular matrix integrity was assessed. In-depth transcriptome analysis was completed to further reveal the mechanisms by which estradiol signals via ERα to modulate mandibular condylar fibrocartilage health. The results from this work provide mechanistic evidence for the role of estradiol on TMJ growth and homeostasis and can be utilized for development of therapeutic targets to inhibit degeneration and promote regeneration of the mandibular condylar fibrocartilage.

## Results

### ERα promotes estrogen-induced changes to cell numbers in the growing mandibular condylar fibrocartilage

**Figure 1:**
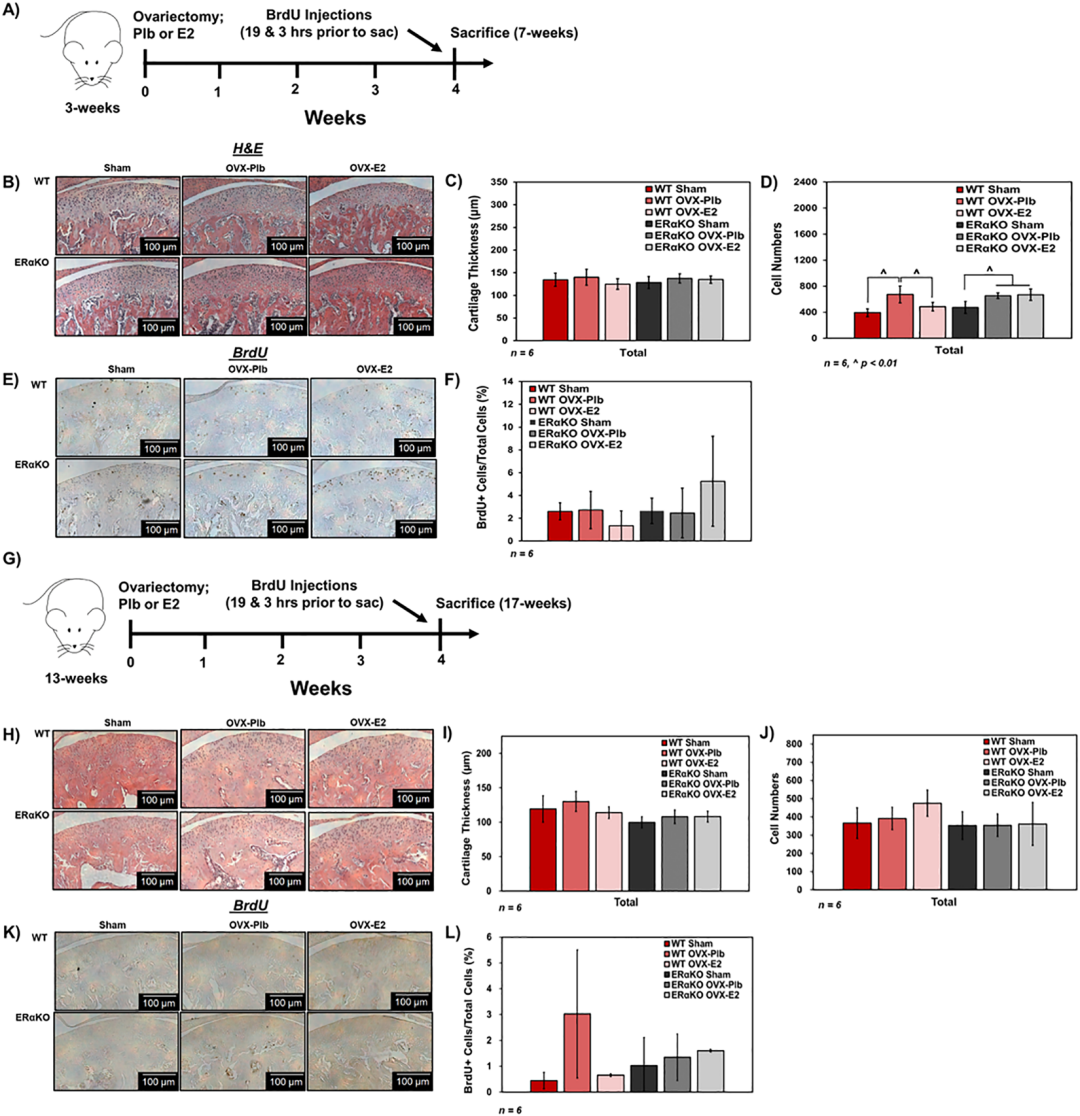
Role of estradiol via ERα on histomorphometry and cell proliferation of mandibular condylar fibrocartilage from 7-week and 17-week old WT and ERαKO female mice after sham, ovariectomy plus placebo treatment, and ovariectomy plus estradiol treatment. Data from 7-week old mice: (**A**) Timeline for experiment (**B**) Representative hematoxylin and eosin images of all groups. (**C**) Cartilage thickness and (**D**). Cell numbers determined by histomorphometry. (**E**) Representative BrdU images illustrating cell proliferation and (**F**). Quantification of percentage of BrdU+ cells. Data from 17-week old mice. (**G**) Timeline for experiment. (**H**) Representative hematoxylin and eosin images of all groups (**I**) Cartilage thickness and (**J**). Cell numbers determined by histomorphometry. (**K**) Representative BrdU images illustrating cell proliferation and (**L**). Quantification of percentage of BrdU+ cells. All values represent means ± standard deviation. n = 6 for all data, ^^^p < 0.01.

Figure 1A illustrates the experimental timeline for OVX, treatment, and age at sacrifice for studies on the growing mandibular condyle. The generation of the homozygous ERα^−/−^ mice results in increased sex steroid levels because of disturbed negative feedback regulation^10^. To avoid confounding endogenous sex steroid effects, all mice were ovariectomized and treated with placebo or estradiol with the sham group serving as the control. Estradiol treatment had no significant effect on fibrocartilage thickness as seen in Fig. 1B,C. However, estradiol treatment resulted in a decrease in total cell numbers compared to placebo treatment in WT mice and no significant change in numbers in ERαKO mice with the same treatment **(**Fig. 1D**)**. These changes in cell numbers may be due to a decrease in cell proliferation with estradiol treatment as shown by the trend in WT samples seen in Fig. 1E,F.

### ERα does not significantly affect cell numbers in the mature mandibular condylar fibrocartilage

The ovariectomy study design for 17-week mice is shown in Fig. 1G. Representative H&E and BrdU images are shown in Fig. 1H,K. Higher magnification BrdU images are included in Supplementary Figure 1. The original hypothesis was that estradiol promoted similar effects in mature condylar fibrocartilage compared to growing fibrocartilage. However, negligible changes to fibrocartilage thickness, cell numbers, and proliferation were observed in tissue from 17-week old mice **(**Fig. 1I,J and L**)**.

### Estradiol via ERα promotes chondrogenesis in 7-week and 17-week mice

**Figure 2:**
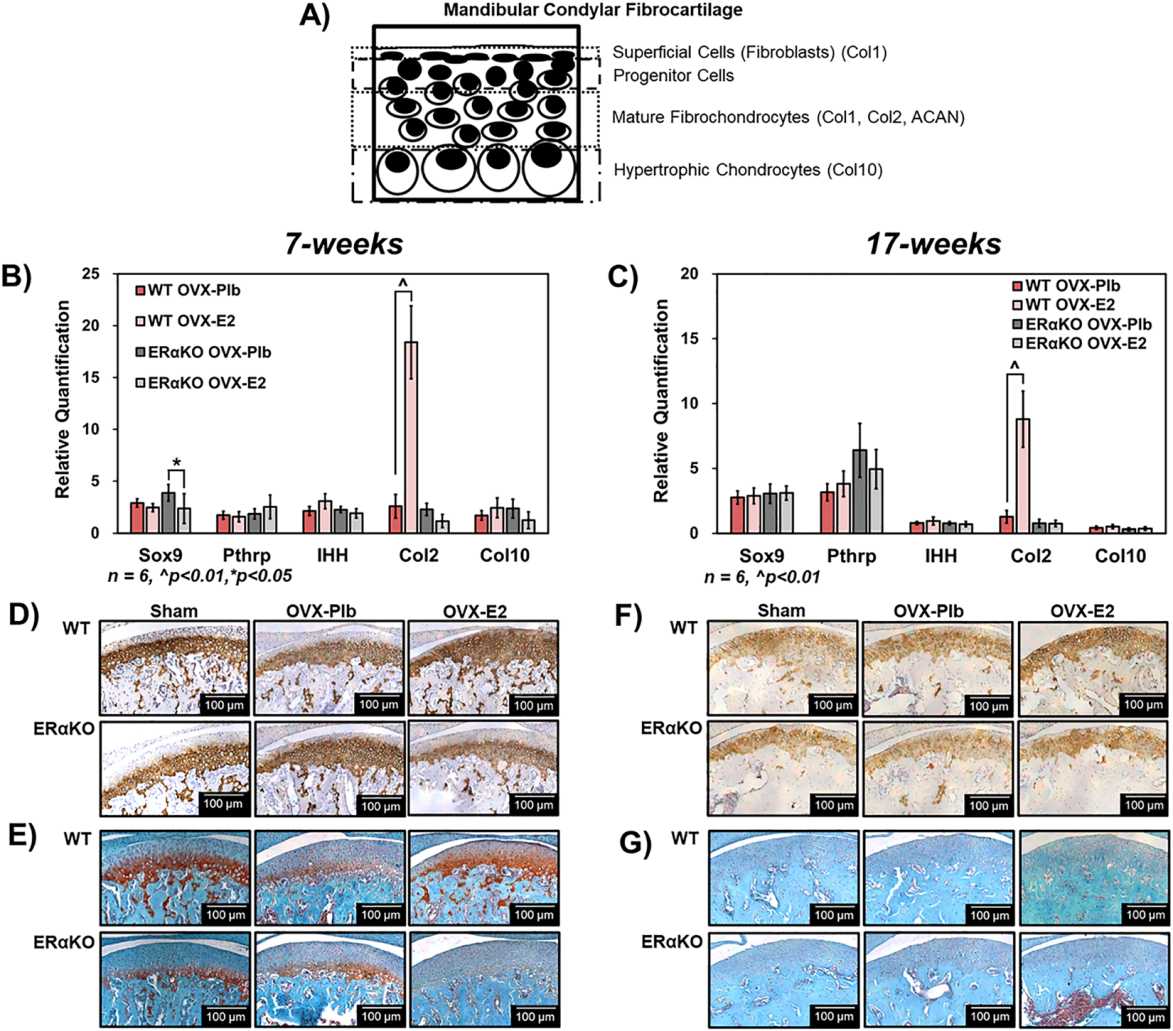
Role of estradiol via ERα on chondrogenesis in the mandibular condylar fibrocartilage of 7-week and 17-week old female mice. (**A**) Schematic illustrating distinct cell phenotypes and respective matrix production in the mandibular condylar fibrocartilage (**B**) qPCR gene expression of Sox9, Pthrp, Ihh, Col2, and Col10 from 7-week old mice (**C**) qPCR gene expression of Sox9, Pthrp, Ihh, Col2, and Col10 from 17-week old mice. (**D**) Representative Col2 immunohistochemical images and (**E**). Representative safranin-O images illustrating proteoglycans from 7-week old mice. (**F**) Representative Col2 immunohistochemical images and (**G**). Representative safranin-O images from 17-week old mice. All values represent means ± standard deviation. n = 6 for all data, ^^^p < 0.01, ^*^p < 0.05.

The role of estradiol via ERα on mandibular condylar fibrocartilage maturation was investigated via extracellular matrix gene expression and protein localization evaluation. The schematic in Fig. 2A illustrates the distinct cells and specific extracellular matrix production within the layers of the mandibular condylar fibrocartilage. Estradiol treatment increased Col2 qPCR gene expression in WT but not ERαKO mice at both ages but did not significantly affect the qPCR gene expression of chondrogenic markers including Sox9, Pthrp, Ihh, and Col10 in either genotype except for a decrease in Sox9 expression in ERαKO mice **(**Fig. 2B,C**)**. Further, estradiol treatment increased Col2 immunostaining and positive staining of the sulfated glycosaminoglycans on the proteoglycans by SafO staining at 7-weeks **(**Fig. 2D,E**)**. The changes in Col2 and SafO staining were not as prevalent in the 17-week samples (Fig. 2F,G**)**.

### Estradiol plays a differential role in gene regulation in growing vs. mature mandibular condylar fibrocartilage

**Figure 3:**
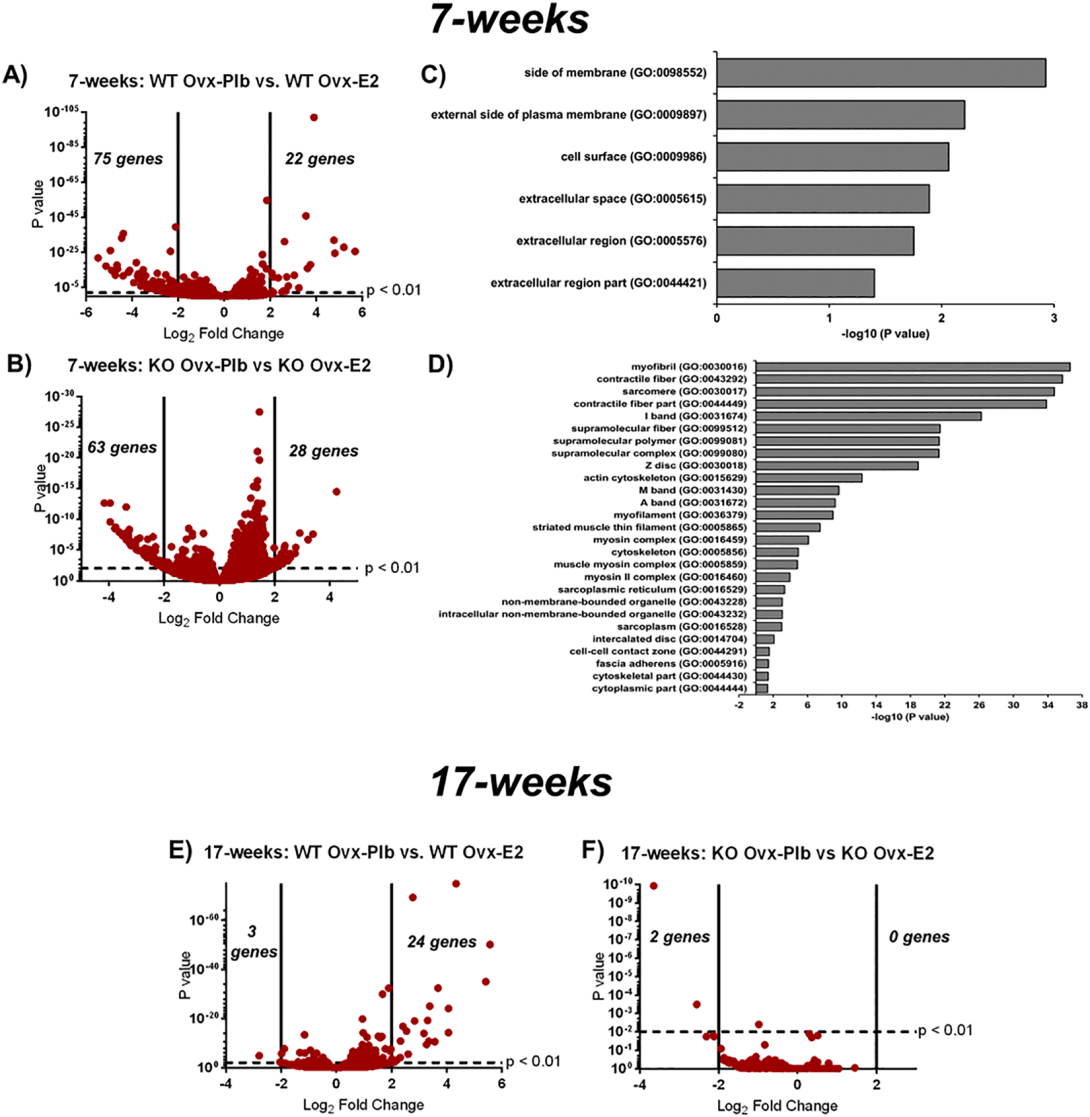
Estradiol via ERα differentially regulates gene expression in 7-week vs. 17-week old mandibular condylar fibrocartilage as determined by RNA sequencing. (**A**) Volcano plot illustrating significant genes as a function as fold change in 7-week WT OVX-Plb vs. OVX-E2 and (**B**) 7-week ERαKO OVX-Plb vs. ERαKO OVX-E2 with significant genes that are regulated at a fold change Log2 > 2 and p < 0.01. (**C**) Significance of biological process via pathway enrichment analysis on significant genes at p < 0.01 via the GO cellular component analysis for 7-weel WT groups and (**D**) 7-week ERαKO groups. (**E**) Volcano plot illustrating significant genes as a function of fold change in 17-week old WT OVX-Plb vs. OVX-E2 and (**F**) 17-week old ERαKO OVX-Plb vs. ERαKO OVX-E2 with significant genes that are regulated at a fold change Log2 > 2 and p < 0.01.

To further decipher the mechanism by which estradiol alters the growing and mature mandibular condylar fibrocartilage, RNA sequencing was conducted to provide a non-biased complete scan of the entire murine genome in response to estradiol treatment in WT and ERαKO mice of both age groups. Volcano plots in Fig. 3 show significant gene expression as a function of fold change. In 7-week old samples, a total of 97 genes are significantly up- or down-regulated at p < 0.01 and log_2_ fold change greater than absolute value of 2 with estradiol treatment in the WT mice as seen in Fig. 3A. In the ERαKO samples, 91 genes are significantly up- or down-regulated with estradiol treatment at p < 0.01 and log_2_ fold change greater than 2 **(**Fig. 3B**)**. Interestingly, only 2 of these genes (Nkg7 and Dbp) are common between the genotypes at 7-weeks indicating other receptors are involved in estradiol-induced changes in the skeletally immature fibrocartilage. Pathway analysis utilizing gene ontology and outputting cellular components resulted in the information shown in Fig. 3C for WT and Fig. 3D for ERαKO. In the growing WT tissue, estradiol via ERα regulates extracellular processes. On the other hand, in the absence of ERα, estradiol regulates several processes with the majority related to muscle cell behavior. Figure 3E illustrates 27 genes are upregulated at p < 0.01 and a log_2_ fold change greater than absolute value of 2 with estradiol treatment in 17-week old WT mice whereas none of these same genes are significantly upregulated in ERαKO samples as seen in Fig. 3F. These volcano plots visually illustrate most of estradiol signaling is via ERα in the mature mandibular condylar fibrocartilage. Also, in comparing the gene regulation between 7-week and 17-week samples in the WT mice, of the 97 genes that are significantly up- or down-regulated with estradiol treatment at 7-weeks, 19 of these are conserved with estradiol treatment at 17-weeks **(**Supplementary Table 5**)**. Further, utilizing the same gene ontology pathway analysis, no pathways were significantly affected by estradiol treatment in 17-week mice of both genotypes. All the genes that were significantly regulated at p < 0.01 and a log2 fold change greater than absolute value of 2 for all the groups are displayed in Supplementary Tables 1–4. Also, a trend of ERα gene expression decreasing with estradiol in both 7- and 17-week WT groups was observed (Supplementary Table 6**)**.

### Estradiol via ERα promotes chondrogenesis in part by inhibiting the canonical Wnt pathway in 7-week tissue

**Figure 4:**
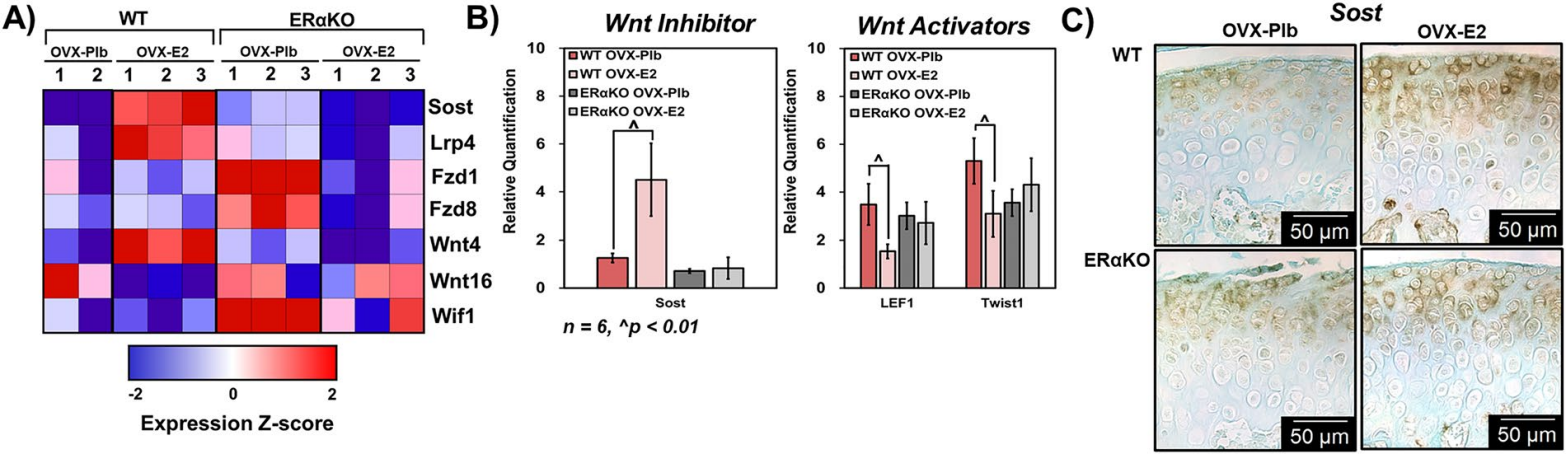
Estradiol via ERα interacts with the canonical Wnt signaling pathway in 7-week old mandibular condylar fibrocartilage. (**A**) Heat map illustrating significant up- and downregulated genes in the canonical Wnt pathway. Z-score values were calculated by subtracting the sample mean from the raw expression value and divided by the standard deviation of the sample (**B**) qPCR gene expression of the Wnt inhibitor sclerostin (Sost) and the Wnt activators lymphoid enhancer binding factor 1 (LEF1) and Twist1. (**C**) Immunohistochemistry of Sost for OVX-Plb and OVX-E2 groups. All values represent means ± standard deviation. n = 6 for PCR datum, ^^^p < 0.01.

Full transcriptome analysis results were utilized to postulate a mechanism by which estradiol may regulate chondrogenesis in the mandibular condylar fibrocartilage. In compiling genes that were significantly regulated at p < 0.05 by estradiol in 7-week old mice, factors from the Wnt signaling pathway were seen to be affected as shown in Fig. 4A. Sclerostin (Sost), LDL receptor related protein 4 (Lrp4), frizzled class receptor 1 (Fzd1), frizzled class receptor 8 (Fzd8), and Wnt4 were upregulated with estradiol treatment in WT mice but not ERαKO as seen in the heat map. On the other hand, Wnt16 and Wnt inhibitory factor 1 (Wif1) were downregulated in the WT samples with estradiol treatment but not the ERαKO tissue. Further, no statistically significant change to factors affecting chondrogenesis including Sox9, Pthrp, Ihh, Runx2, FGF2, Bmp2, TGFβ-2, SMAD4, or Notch1 were found (Supplementary Figure 2). Transcription factors, receptors, and co-factors located within the canonical Wnt pathway were therefore investigated to further determine if estradiol via ERα modulates chondrogenesis by suppression of the Wnt pathway. Estradiol treatment in WT mice increased the qPCR gene expression of the Wnt inhibitor Sost and decreased the Wnt activator LEF1 and downstream mediator Twist1 **(**Fig. 4B**)**. However, estradiol treatment in ERαKO mice had no significant effect on the qPCR gene expression of the Wnt inhibitors or activators examined **(**Fig. 4B**)**. These results were confirmed by immunohistochemical staining of Sost **(**Fig. 4C**)**.

### Estradiol via ERα promotes anabolic gene expression in mature mandibular condylar fibrocartilage

**Figure 5:**
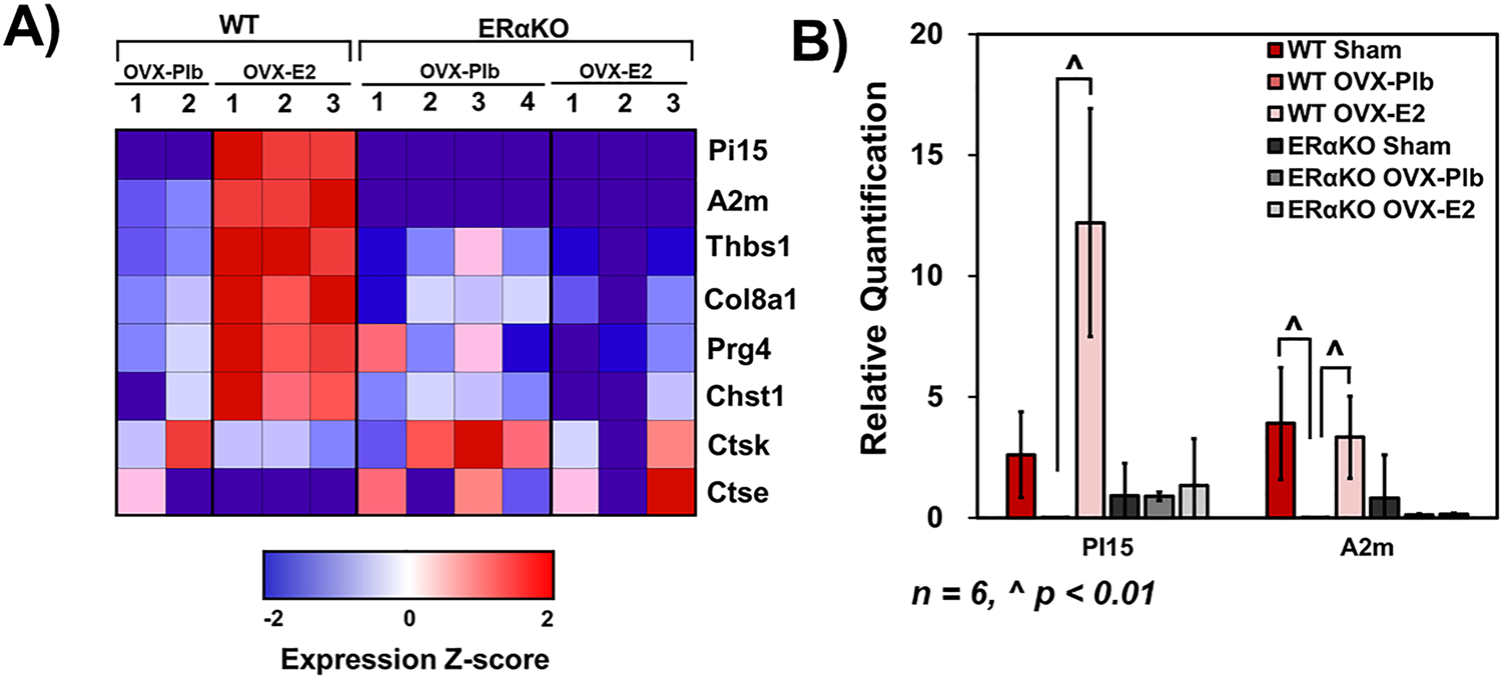
Estradiol via ERα upregulates anabolic extracellular matrix genes and downregulates catabolic genes in 17-week old mandibular condylar fibrocartilage. (**A**) Heat map illustrating upregulation of protease inhibitors and matrix proteins and downregulated of proteases with estradiol treatment in WT but not ERαKO samples. Z-score values were calculated by subtracting the sample mean from the raw expression value and divided by the standard deviation of the sample (**B**) qPCR gene expression of Pi15 an A2m in all WT and ERαKO groups. All values represent means ± standard deviation. n = 6 for PCR datum, ^^^p < 0.01.

In the RNA sequencing analysis, a number of genes that are involved in anabolic processes in the fibrocartilage were upregulated at p < 0.01 as illustrated in the heatmap in Fig. 5A. Specifically, the protease inhibitors alpha-2-macroglobulin (A2m) and protease inhibitor 15 (Pi15) and the extracellular matrix molecules thrombospondin 1 (Thbs1), collagen type 8 alpha 1 chain (Col8a1), proteoglycan 4 (Prg4), and carbohydrate sulfotransferase 1 (Chst1) were all significantly upregulated solely in the WT mice. On the other hand, estradiol treatment significantly downregulated cathepsin K (Ctsk) and cathepsin E (Ctse) in the WT but not ERαKO fibrocartilage. Further analysis of the gene expression using qPCR illustrated a significant increase in Pi15 and A2m expression in WT samples with estradiol treatment and no significant effect in the ERαKO fibrocartilage **(**Fig. 5B**)** indicating the role of ERα in estradiol-based regulation of protease inhibitors.

### Estradiol via ERα decreases protease activity and matrix degradation in 17-week tissue

**Figure 6:**
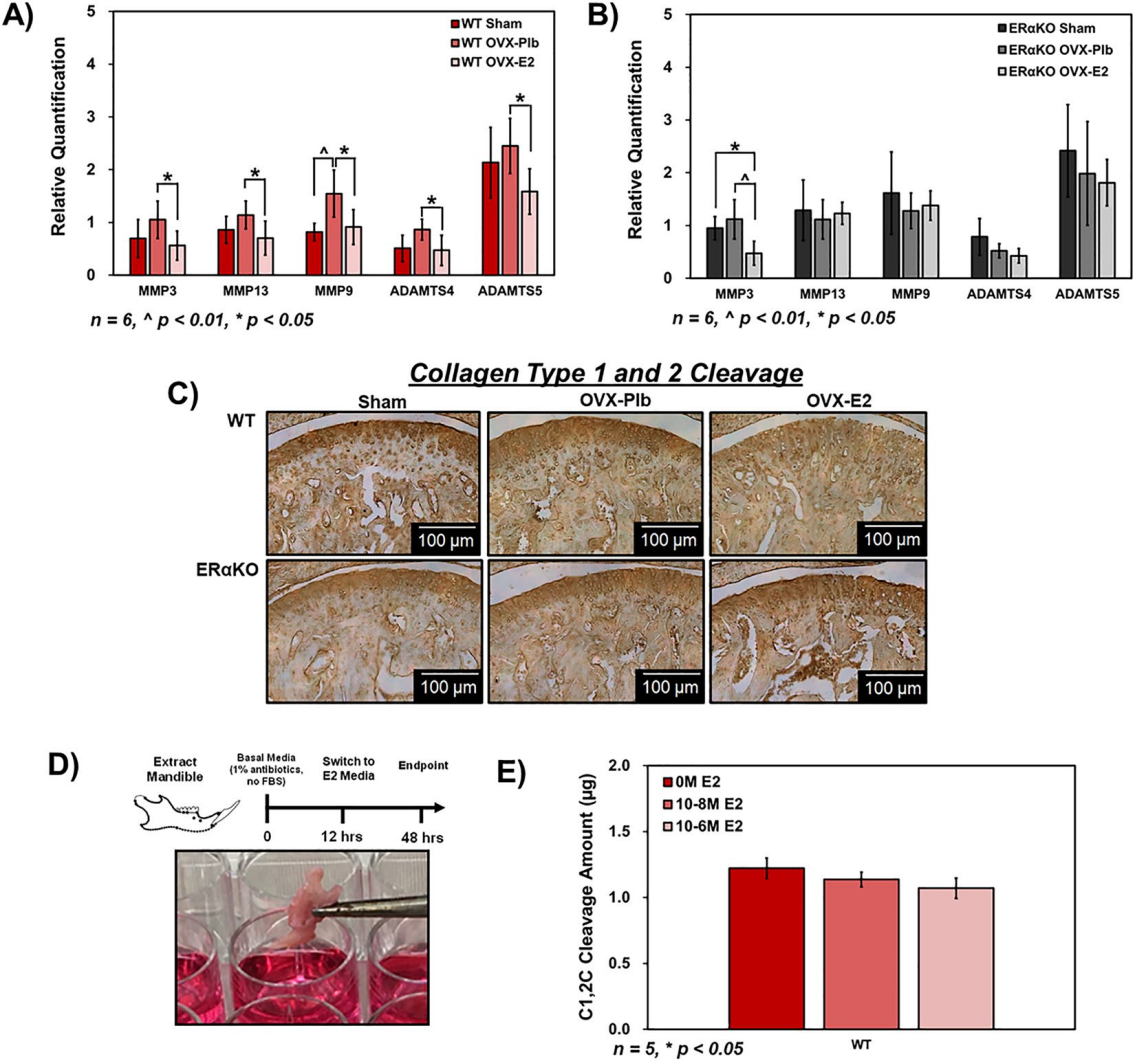
Estradiol via ERα decreases protease activity and collagen cleavage in 17-week old mandibular condylar fibrocartilage. (**A**) qPCR gene expression of proteases MMP3, MMP13, MMP9, ADAMTS4, ADAMTS5 of all WT (**B**) and ERαKO groups. (**C**) Immunohistochemistry of collagen type 1 and 2 cleavage epitopes of WT and ERαKO groups. (**D**) Explant study timeline and digital micrograph illustrating the mandible in culture for 48 hours estradiol treatment. (**E**) Role of estradiol on collagen type 1 and 2 cleavage (C1,2 C) as measured by ELISA in explant culture. All values represent means ± standard deviation. n = 6 for PCR datum and n = 5 for explant datum, ^^^p < 0.01, ^*^p < 0.05.

The role of estradiol on protease expression and collagen cleavage was assessed to determine downstream effects of increased protease inhibitor activity. Estradiol treatment decreased qPCR gene expression of proteases including MMP3, MMP13, MMP9, ADAMTS4, and ADAMTS5 in WT **(**Fig. 6A**)** but not in ERαKO mice except for MMP3 **(**Fig. 6B**)**. Immunostaining of collagen type 1 and 2 cleavage epitopes further illustrated the role the decrease in protease activity exhibits on matrix protein stability as seen in Fig. 6C which shows the decrease in collagen cleavage with estradiol treatment in WT mice compared to placebo treatment. No visible changes were observed in ERαKO mice. An explant culture model was employed to confirm the role of estradiol in protease inhibitor expression and resulting effects to the extracellular matrix. Figure 6D illustrates the timeline and visual of the model. Estradiol treatment for 48 hours resulted in a decrease in Col1 and Col2 cleavage fragments in the supernatant as quantified by an ELISA (Fig. 6E).

### Alpha-2-macroglobulin (A2m) and proteinase 15 (Pi15) are regulated by estradiol and are potential protease inhibitors to treat TMJ-DD

**Figure 7:**
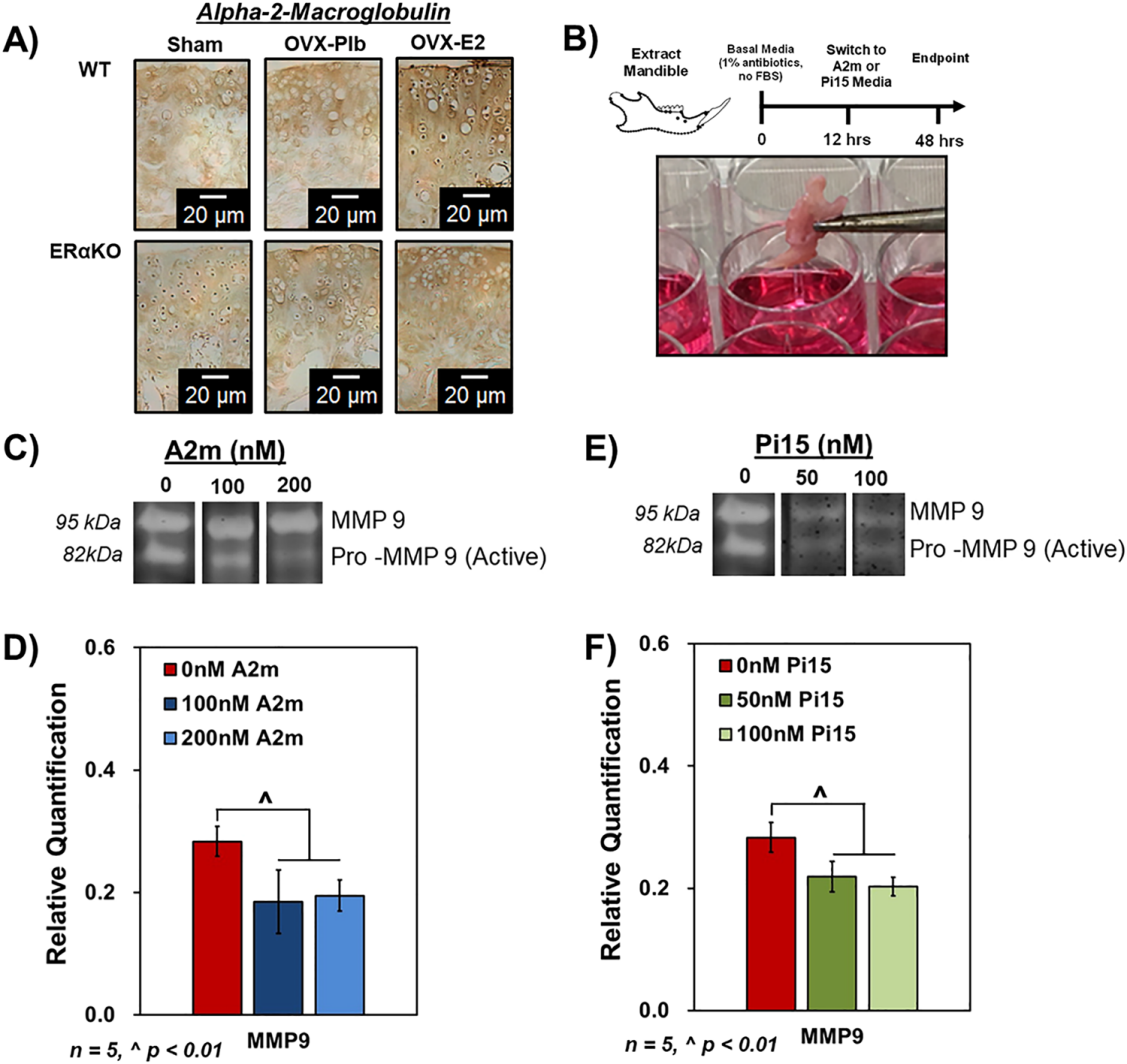
Protease inhibitors alpha-2-macroglobulin (A2m) and proteinase 15 (Pi15) decrease MMP9 activity and expression in 17-week old mandibular condylar fibrocartilage. (**A**) Immunohistochemistry of A2m in all groups. (**B**) Explant study timeline and digital micrograph illustrating the mandible in culture for 48 hours of A2m treatment (0, 100, or 200 nM) or Pi15 treatment (0, 50, or 100 nM). (**C**) Zymogram using 10% gelatin substrate illustrating protease activity as a function of A2m dose (**D**) qPCR gene expression of MMP9 in A2m treatment explant culture. (**E**) Zymogram using 10% gelatin substrate illustrating protease activity as a function of Pi15 dose (**F**) qPCR gene expression of MMP9 in Pi15 treatment explant culture. Zymography gels were cropped and compiled as indicated by the white space separating the groups for increased clarity. Full-length gels are presented in Supplementary Figure 5. All values represent means ± standard deviation. n = 5 for explant culture datum, ^^^p < 0.01, ^*^p < 0.05.

Immunostaining of A2m in WT and ERαKO sections from the OVX experiment illustrates a decrease in protein localization (brown staining) in the OVX group and a recovery with estradiol treatment solely in the WT mice (Fig. 7A). In the A2m and Pi15 dosing experiment utilizing the explant culture model **(**Fig. 7B**)**, A2m, at both 100 and 200 nM, decreased MMP9 activity via zymography analysis (Fig. 7C) and qPCR gene expression **(**Fig. 7D**)**. Similarly, Pi15 treatment at both 50 and 100 nM decreased MMP9 activity **(**Fig. 7E**)** and qPCR gene expression **(**Fig. 7F**)**.

## Discussion

The overarching goal of this study was to determine the role of ERα on the growth and homeostasis of the female mandibular condylar fibrocartilage to provide evidence for the sex predilection of TMJ disease and determine potential mechanisms for healthy joint maintenance. The major findings from this study indicate that estrogen via ERα promotes chondrogenesis by potentially suppressing the canonical Wnt signaling pathway in the mandibular condylar fibrocartilage and maintains homeostasis by upregulating protease inhibitors, thereby decreasing protease activity in the condylar fibrocartilage.

The condylar fibrocartilage of the TMJ is unique compared to other articular cartilages. Specifically, the TMJ fibrocartilage acts like a growth plate cartilage and undergoes endochondral ossification to promote condylar growth^22^. However, the mandibular condylar fibrocartilage does not experience growth plate closure and instead continues to function as an articular cartilage after puberty^3,23^. In growing mice, we found that estrogen replacement caused a decrease in cell numbers in ovariectomized WT but not ERαKO mice. Previously, we have shown that estrogen replacement resulted in similar effects in ERβKO female mice^19^. Together the results suggest that both ERα and ERβ signaling are critical for estrogen’s inhibition of mandibular condylar fibrocartilage growth. Similar results have been reported in the long bones, where both female ERαKO^24^ and ERβKO^25^ mice have increased longitudinal bone growth. However, recent studies have suggested that local cartilage-specific ERα signaling is involved in estrogen’s inhibition of longitudinal bone growth plate cartilage thickness^26^ whereas the effects of ERβ on mediating longitudinal bone growth is unclear^27^. Thus, local administration of ERα-specific agonists may be a novel treatment option in skeletally immature patients with severe mandibular prognathism. On the other hand, we did not observe a significant effect of estradiol on growth in mature mice. Previously, the role of estrogen in skeletally mature female rats utilizing an explant culture model illustrated a decrease in fibrocartilage thickness and cell proliferation similar to young rodents, effects we did not observe in this study^21^. This difference may be due to differences in cell signaling within the *in vivo* OVX model compared to the *in vitro* explant culture model. Our results potentially indicate that targeting ERα with an ERα-specific agonist such as propylpyrazole triol (PPT) in skeletally mature patients may not be effective in inhibiting growth compared to young patients.

Also, it was determined that in the absence of ERα, estradiol promoted myogenesis and/or muscle homeostasis in the growing, female mandibular condylar fibrocartilage whereas estradiol via ERα regulated non-specific extracellular cell processes. Therefore, estrogen treatment in young mice may highlight a potential novel role of ERα signaling in directing the mandibular condylar cartilage progenitor cells down the chondrogenic pathway as opposed to the myogenic pathway^28^. Further, this analysis indicates the multiple receptors in which the estradiol ligand can interact and promote transcription beyond strictly binding to ERα in the growing mandibular condylar fibrocartilage. On the other hand, in the mature mandibular condylar fibrocartilage, all the genes that were up-regulated with estradiol were modulated via ERα suggesting this receptor is the sole mediator of estrogen-induced changes to the mature mandibular condylar fibrocartilage. In young growing mice, the mandibular condylar cartilage contains a large number of fibrocartilage progenitor cells^29^ that decrease in number with age^30^. As such, in the 17-week mice, the role of estrogen in mediating the activity of these progenitor cells may be diminished and only requires ERα for regulation. Overall, our results provide evidence for a differential role that ERα plays in the skeletally immature versus mature mandibular condylar fibrocartilage of female mice.

It is evident from this study that estrogen replacement treatment for four weeks promoted TMJ chondrogenesis. To investigate the mechanism, we assessed the gene expression of a range of the cell fate mediators and transcription factors known to regulate differentiation of the progenitor cells There was no significant change in gene expression of any factors of the TGFβ family, Sox9, Pthrp/Ihh, or Notch that were studied in response to estradiol treatment. Previously, it has been shown that inhibiting the canonical Wnt pathway via Sost promotes chondrogenesis in the mandibular condylar fibrocartilage^29^ whereas an increase in canonical Wnt signaling decreases mandibular condylar fibrocartilage chondrogenesis^31^. In this study, it was determined that estradiol significantly increased Sost mRNA and protein expression in WT but not in ERαKO mice. Further, we determined that phosphorylated (inactive) β-catenin staining was also enhanced with estradiol treatment in WT mice with no observable change in ERαKO mice (Supplementary Figure 3). Previously, it has been shown that postmenopausal women on estrogen replacement therapy had decreased Sost mRNA in bone biopsies from the posterior iliac crest^32^. Further, Galea *et al*. illustrated the role both ERs play in Sost expression in osteoblasts; ERα was shown to maintain the basal expression while ERβ was shown to mediate the acute reduction of Sost expression with increases in estradiol^33^. Results from our laboratory have indicated ERβ plays an insignificant role in the increase in Sost expression from estradiol treatment in the mandibular condylar fibrocartilage^19^. It is probable that estradiol treatment plays a differential role in specific bone cells compared to specific cells in the fibrocartilage. In this study, Sost protein is localized to the superficial and proliferative zones of the mandibular condylar fibrocartilage indicating the cells in these layers (namely the progenitor cells in the proliferative zone) are the cells most heavily expressing and responding to Sost, especially in response to estradiol treatment. Thus, these results suggest estradiol via ERα may promote mandibular condylar chondrogenesis by suppressing the canonical Wnt signaling pathway via Sost. However, in addition to Sost, it is possible that estrogen via ERα directly regulates transcriptional activation of Col2^34^. Therefore, multiple studies are planned with the Sost antibody and the Col2 promoter to delineate the signaling pathway mediating estrogen’s transcriptional regulation of Col2 in the mandibular condylar fibrocartilage.

The existence of a link between estrogen deprivation in post-menopausal women and TMJ-DD highlights the role estrogen may play in homeostasis of the mandibular condylar fibrocartilage. Specifically, clinical data suggests women with a single nucleotide polymorphism in the ERα gene may be at increased risk of developing TMJ-DD^35,36^. In this study, it was observed that estradiol significantly upregulated anabolic genes that function in the extracellular matrix of the articular fibrocartilage. Further, it was seen that estradiol via ERα decreased protease gene expression and cleavage of Col1 and Col2 in the fibrocartilage matrix. Previous characterization of the aging mandibular condylar fibrocartilage has illustrated the necessity of protease activity in the joint to maintain articulation after growth is complete^37^. Our analysis indicates estradiol plays a role in this process. RNA sequencing differential analysis pointed to the upregulation of protease inhibitors as a possible mechanism by which estradiol inhibits protease activity via ERα. Specifically, the protease inhibitors Pi15 and A2m were the two most significantly regulated genes and both shown to reduce MMP9 activity in the mandibular condylar fibrocartilage explant model. Proteinase 15, or peptidase 15, is a gene that encodes a trypsin inhibitor. To our knowledge, there is very limited understanding of Pi15 activity in specific tissues and cells^38^. As such, this presents a unique target to further pursue in homeostasis and degeneration of the mandibular condylar fibrocartilage. Future studies utilizing a wider range of Pi15 peptide concentrations and the recombinant peptide instead of synthetic are necessary to further probe the effect of Pi15 in homeostasis of the mandibular condylar fibrocartilage.

A2m is a large, tetrameric glycoprotein found in plasma of vertebrates and functions as a nonspecific protease inhibitor that has the ability to block proteases from all four classes^39–41^. Cleavage of a unique set of amino acids found near the center of the polypeptide chain known as the “bait” region triggers a conformational change and resulting irreversible entrapment of the protease^40,42^. Consequently, a receptor binding domain is then presented on the surface of the A2m-proteinase unit that signals for cell-mediated endocytosis followed by proteolytic degradation within the lysosomes^40,43–45^. Studies have investigated the role of estrogen or similar molecules on A2m expression. Lim *et al*. illustrated an increase in A2m gene expression with administration of diestylstilbestrol, a synthetic estrogen, in chicken ovaries^46^. Interestingly, treating bovine granulosa cells with A2m results in a dose-dependent increase in estradiol production^47^. Further, the exact mechanism by which estrogen promotes transcription of A2m is not clear. In these studies, we show estradiol treatment increases A2m gene expression in only WT not ERαKO mice at 17-weeks. However, other studies in bone have shown A2m upregulation via estradiol independently of either ERα or ERβ suggesting this is not the sole mechanism^48^. Current studies pursuing the exact receptor and membrane vs. nuclear signaling are underway to pursue the mechanism in more depth.

A2m has been shown previously to be a potential inhibitor of posttraumatic osteoarthritis in the knee^49^. In analyzing synovial fluid and *in vitro* chondrocyte culture from human patients with OA, A2m levels were significantly lower in OA patient synovial fluid and A2m treatment in chondrocyte culture decreased MMP3, MMP9, and MMP13 expression. Currently, the mechanism and timeline for A2m’s effects on downstream gene transcription have not been determined. However, our data illustrating a potential effect of A2m on MMP transcription coupled with past results in chondrocytes provides new avenues of study. Excitingly, a Phase 1/II clinical trial is currently underway investigating A2m for the treatment of tibiofemoral joint pain due to cartilage degeneration, osteoarthritis, inflammation, and meniscal degeneration. These studies are based on autologous A2m obtained from either the patient’s own blood or plasma in a relatively quick procedure at the medical office^50^. Further, recent datum suggests that molecular variants of A2m that can be synthesized prior to surgery are effective at reducing knee osteoarthritis in a rat model and provide an additional option for clinical feasibility^51^. As such, there is potential for similar treatment to reduce TMJ degeneration utilizing A2m.

We recognize every study has limitations. In the studies presented here, only the role of estradiol signaling on the mandibular condylar fibrocartilage was assessed. Insulin growth factor, progesterone, and relaxin levels are altered after ovariectomy and have been shown to affect articular cartilage and subchondral bone^52,53^. Serum levels of these other hormones were not assessed but should be conducted in the future to further ascertain potential effects. Also, the importance of the subchondral bone in the bone-cartilage unit of the TMJ regarding joint health and OA diagnosis has been discussed. Recent studies from Shi *et al*. illustrate the correlation of low condylar bone quality as determined by bone mineral density and bone volume/total volume with increased TMJ-OA and the model as use for diagnostics in the future^54^. However, in these studies, we did not assess changes to the subchondral bone or condyle in response to OVX and estradiol treatment in either WT or ERαKO mice. Future studies must focus on the potential role of estradiol via ERα on the bone of the TMJ. Also, recent evidence from Ucer *et al*. illustrate distinct pathways by which sex steroid deficiency and aging promote bone loss independently^55^. Thus, it is likely that aging-specific mechanisms including mitochondrial dysfunction, reactive oxygen species accumulation, and DNA damage also play a role in mandibular condylar fibrocartilage degeneration independent of estrogen signaling. Lastly, while the role of estrogen via ERα on the articular fibrocartilage of the TMJ was assessed globally, future studies on the role of the receptor in the specific cells using cell-specific conditional knock-outs will be beneficial.

In conclusion, the studies presented here illustrate the probable roles of estrogen via ERα on the growth and homeostasis of the mandibular condylar fibrocartilage of the TMJ. By investigating changes in young mice with growing condyles and comparing to adult mice with mature tissue, the importance of estrogen signaling at different ages was determined. In young tissue, Sost levels may be crucial to maintain healthy fibrocartilage growth and inhibit excess turnover and hypertrophic chondrogenesis. In older tissue, inhibition of protease activity is vital in supporting healthy extracellular matrix tissue that functions to withstand the loads of the TMJ. Both processes are controlled by estrogen via ERα signaling. Thus, targeting ERα with an agonist or delivering a protease inhibitor such as A2m to the mandibular condylar fibrocartilage may be an effective treatment to reduce TMJ-DD in post-menopausal women and partially address one component of the sex dimorphism of TMJ disease.

## Materials and Methods

### Mice

All experiments were performed in accordance with animal welfare based on an approved Institutional Animal Care and Use Committee (IACUC) protocol (#AAAH9166) from Columbia University. Breeding pairs of C57BL/6 ERαKO^-/+^ (Ex3αERKO^56^) heterozygous male and heterozygous females were donated from Dr. Kenneth Korach at the National Institute of Environmental Health Sciences at National Institutes of Health to breed female WT and ERαKO mice. Total ERα−/− mice have increased sex steroid levels because of disturbed negative feedback regulation^10^. Thus, to avoid confounding endogenous sex steroid effects, all mice were ovariectomized and treated with placebo or estradiol to determine the effect of estradiol via ERα. WT and ERαKO female mice were divided into three groups: sham, ovariectomy with placebo treatment, and ovariectomy with estradiol treatment (for all conditions: n = 6 for RTPCR mRNA and n = 6 for histology). To determine effects in the growing mandibular condylar fibrocartilage, 3-week old mice were treated for 4 weeks with placebo or estradiol. For the effects on the mature fibrocartilage, 13-week old mice were treated for 4 weeks. Mice were administered placebo or 17β-estradiol (0.01 mg/pellet for 60-day release, Innovative Research of America, FL) for 4 weeks at a daily dose of 11 ng/g body weight for 7-week old mice (assuming 15 g body weight) and 7 ng/g body weight for 17-week old mice (assuming 25 g body weight). Doses similar to that used in this study resulted in serum estradiol concentrations of roughly 600 pM^48^. Normal serum levels of estradiol are between 70 and 110 pM during diestrus and between 350 and 730 pM during estrus of the female mouse estrous cycle^57^. Thus, the estradiol treatment in this study likely resulted in levels comparable to what is experienced during estrus. Further, the dose of estradiol utilized in these study matches the dose found effective to restore MCC thickness in female, ovariectomized WT mice of the same age^58^. Following 4 weeks of treatment, mice were injected intraperitoneally with 0.1 mg bromodeoxyuridine (BrdU) per gram body weight at both nineteen and three hours prior to euthanasia to track proliferating cells. Body and uterus weights were measured post sacrifice (data not included). For RNA sequencing, WT and ERαKO mice at 6- and 16-weeks were ovariectomized and treated with placebo or 17β-estradiol (0.01 mg/pellet for 60-day release, Innovative Research of America, FL) for 1 week at doses detailed above (n = 2 for WT OVX-placebo, n = 3 for all other groups). Lastly, WT mice at 13-weeks were utilized for the explant culture study (n = 5 for all groups).

### Histology and Histomorphometry

Histomorphometry techniques were employed to determine the effect of estradiol treatment via ERα on mandibular condylar fibrocartilage morphology. Whole mouse heads were sectioned into halves, fixed in 10% formalin for 4 days at room temperature and decalcified in 14% ethylenediaminetetraacetic acid (EDTA) (pH 7.1) (Sigma, St. Louis, MO, USA) for 28 days. Subsequently, the samples were processed through progressive concentrations of ethanol, cleared in xylene, and embedded in paraffin. Sagittal serial sections of 5 μm thickness were made of the TMJ utilizing a Microm HM 355 s microtome (Thermo Fisher Scientific, Waltham, MA, USA). Sections representing the mid-coronal portion of the mandibular head were stained with hematoxylin and eosin (H&E) and Safranin-O (SafO) and used as the representative central section for analysis.

Histomorphometry measurements were made in a blinded, nonbiased manner using the BioQuant computerized image analysis system (BioQuant, Nashville, TN, USA). Analysis was performed on H&E sagittal sections corresponding to the mid-coronal portion of the mandibular condylar head. Average thicknesses were determined for the total cartilage thickness (representative region shown in Supplementary Figure 4). ImageJ was utilized to determine total cell numbers within the outlined cartilage region. Condyles from six mice within each group were analyzed and the average of three-five sections was taken for each sample.

### Immunohistochemistry

For immunohistochemistry, tissue sections were deparaffinized with xylene and rehydrated in progressive ethanol/water solutions with increasing concentrations of deionized water. Following rehydration, the sections were digested for 10 minutes with pepsin (Lab Vision, Fremont, CA, USA) at 37 °C (Col2 and C1,2 C antibodies) or 0.01 M citrate buffer (pH = 6.0, Sigma) for 5 minutes at 60 °C (Sost, A2m, and Pi15) for unmasking, washed with PBS, and treated with a 3 vol% hydrogen peroxide in methanol solution for 10 minutes at room temperature to inhibit endogenous peroxidase activity. All sections were blocked with 10% normal goat serum (Life Technologies) to reduce non-specific binding of the antigen with the primary antibody. Immunohistochemical staining was performed using the SuperPicture™ Polymer HRP Broad Spectrum Detection Kit (Life Technologies) following the procedure recommended by the manufacturer. Further details are in the Supplementary Materials and Methods.

### mRNA Extraction and PCR Amplification for Gene Expression

After duration of treatment, mRNA from the condylar cartilage of all groups was extracted to analyze the effects of estradiol on chondrogenic markers and proteases via ERα. For each mouse, the MCC (left and right) was carefully isolated from all other soft tissue and dissected under a dissecting microscope. mRNA was extracted with TRIzol Reagent (Ambion by Life Technologies) following the manufacturer’s protocol and treated with DNase treatment and removal kit (Ambion, Life Technologies) to remove any residual DNA contaminants. Reverse transcription was performed to convert mRNA to cDNA utilizing the High Capacity cDNA Reverse Transcription Kit (Applied Biosystems, Foster City, CA) in RNase-free conditions following the manufacturer’s protocol. Real-time polymerase chain reaction (RT-PCR) was conducted to assess the relative levels of genes of interest using the ViiA™ 7 Real-Time PCR System (Applied Biosystems, Life Technologies) following the protocol detailed in Chen *et al*.^19^. Expression of each gene of interest was determined relative to the Gapdh housekeeping gene (Gapdh – MM99999915_g1) utilizing the ΔΔC_T_ method. Gene expression was analyzed for the following markers: collagen type 2 (*Col 2a1* – Mm00491889_m1), collagen type 10 (*Col 10a1* – Mm00487041_m1), SRY-box containing gene 9 (*Sox9* – MM00448840_m1), parathyroid hormone-related peptide (*PTHrP* – Mm00436057_m1), indian hedgehog (*Ihh* – Mm00439613_m1), runt-related transcription factor 2 (*Runx2* – Mm00501578_m1), fibroblast growth factor 2 (*FGF2 –* Mm00433287_m1), bone morphogenetic protein 2 (*BMP2* – Mm01340178_m1), transforming growth factor beta – 2 (*TGFβ-2* – Mm00436955_m1), mothers against decapentaplegic homolog 4 (*SMAD4* – Mm03023996_m1), translocation-associated notch homolog 1 (*Notch1* – Mm00627185_m1), sclerostin (*Sost* – Mm00470479_m1), lymphoid enhancer-binding factor 1 (*LEF1* – Mm00550265_m1), twist-related protein 1 (*Twist1* – Mm00442036_m1), protease inhibitor 15 (*Pi15* – Mm00499734_m1), alpha-2-macroglobulin (*A2m* – Mm00558642_m1), matrix metalloproteinase 3 (*MMP3 –* Mm00440295_m1), matrix metalloproteinase 13 (*MMP13 –* Mm00439491_m1), matrix metalloproteinase 9 (*MMP9 –* Mm00442991_m1), aggrecanase-1 (*ADAMTS4 -* Mm00556068_m1), and aggrecanase-2 (*ADAMTS5 –* Mm00478620_m1). All primers were purchased from Applied Biosystems.

### RNA-Sequencing, Library Generation, and Bioinformatics Analysis

RNA was extracted and purified from mandibular condylar fibrocartilage cells utilizing PureLink™ RNA Mini Kit (Invitrogen, ThermoFisher Scientific) and following the manufacturer’s protocol. Volcano plots illustrating gene significant values as a function of log2 fold change were generated using GraphPad Prism. Selected genes that were differentially regulated in response to estradiol treatment with an adjusted p < 0.01 were analyzed utilizing the PANTHER overrepresentation test (release 20170413) and the GO Ontology database (released 2017-04-24) to determine the biological processes affected by estradiol treatment. Heat maps illustrating the relative gene expression were fabricated utilizing Gene Pattern from the Broad Institute and represent z-score values calculated by subtracting the sample mean from the raw expression value and then dividing by the standard deviation of the sample. Additional details can be found in the Supplementary Materials and Methods.

### Mandible Explant Cultures

Explant cultures were utilized to further investigate the role of both estradiol and A2m on condylar fibrocartilage degradation. Entire mandibles with condyle and condylar fibrocartilage intact were harvested from 13-week old WT female mice and cultured in a 24-well plate with 1.5 mL of FBS-free BGJb medium supplemented with 1% Penicillin-Streptomycin (ThermoFisher Scientific) overnight. Following overnight incubation, media was removed, samples were washed 2 × with sterile PBS, and fresh media with either 0 M, 10^−8^ M, or 10^−6^ M 17β-estradiol (17β-estradiol, Sigma E2758, for the estradiol study), 100 nM and 200 nM A2m (from human plasma, Sigma SRP6314), or 50 nM and 100 nM Pi15 (synthetic – based on human peptide sequence, abcam ab23016) was added and incubated for 48 hours. Estradiol concentrations were chosen based on similar studies^21^ and further substantiated by evidence of circulating estrogen levels in humans^59^. A2m concentrations were determined based on previous evidence in a knee osteoarthritis model^49^. After 48 hours of treatment, supernatant was collected for zymogram assays and collagen type I and II cleavage ELISAs. Messenger RNA from both left and right samples was pooled together and utilized for RTPCR. For gelatin zymography and ELISA analysis, supernatant from only the left specimen was utilized.

### Zymography

Matrix metalloprotease (MMP) activity in response to A2m treatment to mandible explant cultures was assessed using gelatin zymography. After 48 hours of treatment in explant culture, supernatant (1.5 mL) was aspirated from the samples, concentrated utilizing a centrivap benchtop vacuum concentration (Labconco), reconstituted in 150 µL BGjb medium, and stored in −80 C until used. Tris-glycine gels (10%) with 0.1% gelatin incorporated as a substrate (ThermoFisher Scientific) were utilized and zymography run according to manufacturer’s protocol.

### Collagen Type 1 and 2 Cleavage ELISA

After 48 hours of treatment in explant culture, supernatant (1.5 mL) was aspirated from the samples, concentrated utilizing a centrivap benchtop vacuum concentration (Labconco), reconstituted in 150 µL BGjb medium, and stored in −80 C until used. Detection of collagen type 1 and 2 cleavage fragments in explant culture were measured using a C1,2 C ELISA kit (IBEX Pharmaceuticals Inc.) according to manufacturer’s instructions.

### Statistical Analysis

Values are presented as the mean ± standard deviation. Statistical significance of differences among means was determined by one-way analysis of variance (ANOVA) for protease gene expression datum or two-way ANOVA for all other data to account for genotype effects (WT vs. ERαKO). Post hoc analysis by the Bonferonni method using GraphPad Prism 7.03 was conducted for all data sets. Statistical significance was defined as either *p < *0*.*05* or ^*p* < *0*.*01* as indicated in the figure legends.

### Data Availability

All data generated and analyzed during this study are either included in this publication and the Supplementary Information, accessible through the NCBI GEO database (GSE110304), or available from the corresponding author upon reasonable request.

## Electronic supplementary material

Supplementary Information
