# Supplementary material for: Estrogen Promotes Mandibular Condylar Fibrocartilage Chondrogenesis and Inhibits Degeneration via Estrogen Receptor Alpha in Female Mice
Source: Sci Rep. 2018 Jun 4;8:8527. doi: 10.1038/s41598-018-26937-w (PMC5986784; doi:10.1038/s41598-018-26937-w)
Supplement: Supplementary file 1 — Supplementary Information [file 41598_2018_26937_MOESM1_ESM.pdf]

Supplementary Information

Estrogen Promotes Mandibular Condylar Fibrocartilage Chondrogenesis and Inhibits Degeneration via Estrogen Receptor Alpha in Female Mice

***Jennifer L. Robinson, PhD**[a,c]**,** Paola Soria, DDS[b], Manshan Xu, MD, PhD[b], Mark Vrana[a], Jeffrey Luchetti[a], Helen H. Lu, PhD[c], Jing Chen, DDS, PhD[b], Sunil Wadhwa, DDS, PhD[b]

Columbia University [a]College of Dental Medicine - [b]Division of Orthodontics,

622 West 168th Street

New York, NY 10032

Columbia University [c]Department of Biomedical Engineering

351 Engineering Terrace

1210 Amsterdam Avenue

New York, NY 10027

Manuscript Submitted to ***Scientific Reports***

Corresponding author:
Dr. Jennifer Robinson
Department of Chemical and Petroleum Engineering
1520 West 15th Street, Learned 4132F
Lawrence, KS 66045
P: (785) 864-0298
jlrobinson@ku.edu
Jennyholmrobinson@gmail.com

SUPPLEMENTARY MATERIALS AND METHODS

RNA-Sequencing, Library Generation, and Bioinformatics Analysis

Endogenous DNAse was removed utilizing the DNAse treatment removal kit (Ambion, Life Technologies). Purity and integrity was confirmed using an Agilent 2100 Bioanalyzer (Agilent Technologies) and all values were above 9.0. Libraries were generated for sequencing using an Illumina TruSeq RNA prep kit and sequenced utilizing Illumina HiSeq2500/HiSeq4000 at the Columbia Genome Center. RTA (Illumina) was utilized for base calling and bcl2fastq2 (version 2.17) used for converting BCL to fastq format, coupled with adaptor trimming. The reads were mapped to a reference genome (Mouse: UCSC/mm9) using Tophat (version 2.1.0) with 4 mismatches and 10 maximum multiple hits. Differentially expressed genes were determined using DEseq, an R package based on a negative binomial distribution that model the number reads from RNA seq experiments and tests for differential expression. All raw and processed data for both 7- and 17-week data sets are uploaded to the NCBI GEO database.

Immunohistochemistry and BrdU Staining

Collagen type II (Millipore; MAB8887 – mouse monoclonal (6B3), 1:100 dilution), Sclerostin (Sost) (Abcam; ab115568 – rabbit polyclonal, 1:100 dilution), Alpha-2-Macroglobulin (A2m) (Biorbyt, orb2676 - rabbit polyclonal, 1:100 dilution), Pi15 (Biorbyt, orb375374 – goat polyclonal, 1:100 dilution) and collagen type 1 and 2 cleavage (C1,2C) (IBEX Pharmaceuticals, #50-1035 – rabbit polyclonal, 1:100) were the primary antibodies utilized in this study. After incubation with the primary antibodies (Col2: 60 minutes at RT; Sost, A2m, and C1,2C: overnight at 4°C), sections were washed twice in PBS. The secondary antibody – horseradish peroxidase (HRP) conjugate (SuperPicture™, Life Technologies) was added and incubated for 30 minutes at RT. Following 2X PBS wash, sections were stained with DAB chromogen (30 µL DAB in 1 mL diluent, ImmPACT DAB, Vector Laboratories) for 2 minutes for Col2 and C1,2C sections and 10 minutes for Sost, A2m, and Pi15 samples. For Col2 and Sost staining, sections were then counterstained with either hematoxylin (Col2 staining) or 0.2% Fast Green (Sost staining) for 30 seconds, dehydrated, and mounted.

BrdU immunohistochemical analysis to determine proliferating cells was completed using a BrdU staining kit following the manufacturer's instructions (Zymed Laboratories-Invitrogen

Corporation, Carlsbad, CA, USA). To quantify BrdU, the labeling index (number of BrdU positive cells divided by the total number of cells) was calculated. Three to six sections, corresponding to the same anatomical region utilized to determine total cell number (mid-coronal), were counted for each group and the average index of these sections was used for the labeling index.

<u>Supplementary Table 1:</u> List of genes with corresponding Log2 fold change > absolute value of 2 and p-value < 0.01 from 7-week WT RNA sequencing analysis

Gene	Gene Description	Fold Change (Log2)	P Value
Trpc6	Transient Receptor Potential Cation Channel Subfamily Member 6	3.91	7.87E-103
Pi15	Peptidase Inhibitor 15	3.56	1.38E-46
Cxcl12	C-X-C Motif Chemokine Ligand 12	-2.11	2.44E-40
Spib	Spi-B Transcription Factor	-4.38	1.43E-36
Myb	MYB Proto-Oncogene, Transcription Factor	-4.46	5.06E-34
Lgi2	Leucine Rich Repeat LGI Family Member 2	4.77	1.02E-32
A2m	Alpha-2-Macroglobulin	2.62	6.03E-32
Greb1	Growth Regulation by Estrogen in Breast Cancer 1	5.20	1.22E-28
Prtn3	Proteinase 3	-4.94	7.62E-27
Limch1	LIM and Calphonin Homology Domains 1	-2.33	2.00E-26
Sftpa1	Surfactant Protein A1	5.69	2.70E-26
Asb4	Ankyrin Repeat and SOCS Box Containing 4	4.81	3.15E-25
Ctsg	Cathepsin G	-5.48	1.26E-22
Plac8	Placenta Specific 8	-3.82	5.62E-20
Shank2	SH3 and Multiple Ankyrin Repeat Domains 2	3.75	8.71E-19
Cecr2	Histone Acetyl-Lysine Reader	-4.65	1.33E-18
Alox15	Arachidonate 15-Lipoxygenase	-5.14	4.88E-18
Myo5c	Myosin VC	3.62	1.04E-16
Rag1	Recombination Activating 1	-4.63	1.97E-16
Cd79a	CD79a Molecule	-3.52	6.85E-16
Ms4a3	Membrane Spanning 4-Domains A3	-4.89	1.46E-15
Vpreb3	V-Set Pre-B Cell Surrogate Light Chain 3	-4.11	1.46E-15
St8sia2	ST8 Alpha-N-Acetyl-Neuraminide Alpha-2,8-Sialytransferase 2	-4.16	1.69E-14
Gbp4	Guanylate Binding Protein 4	2.08	5.32E-14
Pou2af1	POU Class 2 Associating Factor 1	-3.59	5.64E-14
Nup210	Nucleoporin 210	-2.90	6.94E-14
Fcrla	Fc Receptor Like A	-3.75	1.62E-13
Csf3r	Colony Stimulating Factor 3 Receptor	-2.54	5.18E-13
Prss34	Protease, Serine 34	-4.73	7.46E-13
Gbp8	Guanylate Binding Protein 8	3.05	9.21E-13
Adipoq	Adiponectin, C1Q and Collagen Domain Containing	-3.42	1.82E-12
Dntt	DNA Nucleotidylexotransferase	-4.40	1.99E-12
Chl1	Cell Adhesion Molecule L1 Like	-3.76	2.72E-12
Mrvi1	Murine Retrovirus Integration Site 1 Homolog	2.11	3.88E-12

4833424O15Rik	PLPPR5, Phospholipid Phosphatase Related 5	2.14	7.78E-12
Gbp6	Guanylate Binding Protein 6	2.71	9.33E-12
Mgl2	Macrophage Galactose N-Acetyl-Galactosamine Specific Lectin 2	2.35	2.80E-11
Ppbp	Pro-Platelet Basic Protein	-3.49	3.50E-11
Cd19	CD19 Molecule	-2.38	3.67E-10
Slfn1	Schlafen 1	-3.62	1.06E-09
Ptprcap	Protein Tyrosine Phosphatase, Receptor Type C Associated Protein	-3.08	1.34E-09
Luzp2	Leucine Zipper Protein 2	-2.28	2.03E-09
Fam129c	Family with Sequence Similarity 129 Member C	-2.23	3.37E-09
Sell	Selectin L	-2.85	3.89E-09
Siglecg	Sialic Acid Binding Ig-like Lectin G	-2.89	8.54E-09
Cd79b	CD79b Molecule	-2.70	8.73E-09
Fcnb	Ficolin 1	-3.93	1.65E-08
Cxcr2	C-X-C Motif Chemokine Receptor 2	-2.92	8.54E-08
Igll1	Immunoglobulin Lambda Like Polypeptide 1	-3.26	1.41E-07
1810033B17Rik	MCEMP1, Mast Cell Expressed Membrane Protein 1	-3.33	1.83E-07
Ly6d	Lymphocyte Antigen 6 Family Member D	-2.87	2.29E-07
Mzb1	Marginal Zone B and B1 Cell Specific Protein	-3.49	6.96E-07
Coch	Cochlin	2.79	1.22E-06
Mcpt8	Mast Cell Protease 8	-3.63	1.24E-06
Ccr2	C-C Motif Chemokine Receptor 2	-2.10	1.95E-06
Dpp4	Dipeptidyl Peptidase 4	-2.40	3.06E-06
Fcho1	FCH Domain Only 1	-2.94	1.04E-05
Ear6	Eosinophil-Associated, Ribonuclease A Family, Member 6	-3.39	1.05E-05
Il1f9	Interleukin 36, Gamma	-3.38	1.35E-05
Fam196b	Family with Sequence Similarity 196 Member B	3.25	1.69E-05
Treml2	Triggering Receptor Expressed on Myeloid Cells Like 2	-2.48	2.01E-05
Camp	Cathelicidin Antimicrobial Peptide	-2.78	2.18E-05
Gpr88	G Protein-Coupled Receptor 88	-2.01	2.65E-05
Pglyrp1	Peptidoglycan Recognition Protein 1	-2.62	2.67E-05
Ly6c2	Lymphocyte Antigen 6 Complex, Locus C2	-3.15	3.12E-05
Prss57	Protease, Serine 57	-3.24	3.12E-05
Padi4	Peptidyl Arginine Deiminase 4	-2.85	8.85E-05
Cd177	CD177 Molecule	-2.58	1.92E-04
Gapt	GRB2 Binding Adaptor Protein, Transmembrane	-2.72	1.99E-04
Cebpe	CCAAT/Enhancer Binding Protein Epsilon	-2.98	2.57E-04
Bcl11a	B-Cell CLL/Lymphoma 11A	-2.45	2.92E-04
Napsa	Napsin A Aspartic Peptidase	-2.37	4.34E-04
Il7r	Interleukin 7 Receptor	-2.39	6.41E-04
Hcn4	Hyperpolarization Activated Cyclic Nucleotide Gated Potassium Channel 4	2.60	6.54E-04
Trem3	Triggering Receptor Expressed on Myeloid Cells 3	-2.78	7.29E-04

Pik3c2g	Phosphatidylinositol-4-Phosphate 3-Kinase Catalytic Subunit Type 2 Gamma	-2.42	8.53E-04
Tbc1d10c	TBC1 Domain Family Member 10C	-2.07	1.11E-03
Ripk4	Receptor Interacting Serine/Threonine Kinase 4	2.68	1.17E-03
Ppef1	Protein Phosphatase with EF-Hand Domain 1	2.07	1.49E-03
Ctse	Cathepsin E	-2.69	1.52E-03
Nfe2	Nuclear Factor, Erythroid 2	-2.26	1.71E-03
Klk4	Kallikrein Related Peptidase 4	-2.69	1.76E-03
Prg3	Proteoglycan 3, Pro-Eosinophil Major Basic Protein 2	-2.62	2.67E-03
Rag2	Recombination Activating 2	-2.60	3.08E-03
Celf6	CUGBP Elav-Like Family Member 6	2.51	3.63E-03
Il2ra	Interleukin 2 Receptor Subunit Alpha	-2.49	3.70E-03
Nkg7	Natural Killer Cell Granule Protein 7	-2.41	4.39E-03
Mapt	Microtubule Associated Protein Tau	-2.14	4.85E-03
Bfsp2	Beaded Filament Structural Protein 2	-2.51	5.71E-03
Vpreb1	V-Set Pre-B Cell Surrogate Light Chain 1	-2.27	5.71E-03
Prg2	Proteoglycan 2, Pro Eosinophil Major Basic Protein	-2.50	5.98E-03
Cyp11a1	Cytochrome P450 Family 11 Subfamily A Member 1	2.14	6.09E-03
Blk	BLK Proto-Oncogene, Src Family Tyrosine Kinase	-2.05	7.16E-03
S100a8	S100 Calcium Binding Protein A8	-2.41	7.22E-03
Dbp	D-Box Binding PAR BZIP Transcription Factor	-2.46	7.26E-03
Retnlg	Resistin Like Gamma	-2.39	8.69E-03
Tmprss3	Transmembrane Protease, Serine 3	-2.40	9.47E-03

Supplementary Table 2: List of genes with corresponding Log2 fold change > absolute value of 2 and p-value < 0.01 from 7-week ERαKO RNA sequencing analysis

Gene	Gene Description	Fold Change (Log2)	P Value
Eef1a2	Eukaryotic Translation Elongation Factor 1 Alpha 2	-3.96	2.21E-13
Obscn	Obscurin, Cytoskeletal Calmodulin and Titin-Interacting RhoGEF	-3.38	9.99E-13
Mb	Myoglobin	-3.76	1.94E-09
Myh2	Myosin Heavy Chain 2	-3.79	3.26E-09
Klhl41	Kelch Like Family Member 41	-3.26	4.66E-09
Acta1	Actin, Alpha 1, Skeletal Muscle	-3.63	1.21E-08
Nkg7	Natural Killer Cell Granule Protein 7	2.91	1.70E-08
Csrp3	Cysteine and Glycine Rich Protein 3	-3.41	1.96E-08
Mcpt8	Mast Cell Protease 8	3.39	2.65E-08
Trdn	Triadin	-3.30	3.13E-08
Mybpc1	Myosin Binding Protein C, Slow Type	-3.51	4.37E-08

Myoz2	Myozenin 2	-3.38	4.63E-08
Lingo1	Leucine Rich Repeat and Ig Domain Containing 1	-2.40	7.02E-08
Tnnt3	Troponin T3, Fast Skeletal Type	-3.33	8.48E-08
Casq1	Calsequestrin 1	-2.89	1.10E-07
Ckm	Creatine Kinase, M-Type	-3.37	1.63E-07
Hfe2	Hemochromatosis Type 2 (Juvenile)	-3.28	1.66E-07
Ldb3	LIM Domain Binding 3	-2.60	1.71E-07
Odam	Odontogenic, Ameloblast Associated	3.21	2.00E-07
Tnnc2	Troponin C2, Fast Skeletal Type	-3.37	2.01E-07
Cacna1s	Calcium Voltage-Gated Channel Subunit Alpha1 S	-2.98	2.04E-07
Cox8b	Cytochrome C Oxidase Subunit 8B, Pseudogene	-3.30	4.32E-07
Ckmt2	Creatine Kinase, Mitochondrial 2	-3.26	7.82E-07
Neb	Nebulin	-3.21	1.03E-06
Tcap	Titin-Cap	-3.19	1.21E-06
Lmod2	Leiomodin 2	-3.04	1.43E-06
Xirp2	Xin Actin Binding Repeat Containing 2	-3.22	1.52E-06
Actn2	Actinin Alpha 2	-3.19	1.90E-06
Gca	Grancalcin	2.38	1.99E-06
Gm129	Gene Model 129	-2.31	2.91E-06
B430306N03Rik	RIKEN cDNA B430306N03 gene	2.35	3.63E-06
Clec4e	C-Type Lectin Domain Family 4 Member E	2.77	3.72E-06
Alpk3	Alpha Kinase 3	-2.69	5.28E-06
Cox6a2	Cytochrome C Oxidase Subunit 6A2	-2.56	8.27E-06
Smpx	Small Muscle Protein, X-Linked	-3.01	8.27E-06
Mylk2	Myosin Light Chain Kinase 2	-2.68	8.62E-06
Jsrp1	Junctional Sarcoplasmic Reticulum Protein 1	-2.78	1.06E-05
Ms4a4c	Membrane Spanning 4-Domains A4A	2.35	1.06E-05
Smtnl1	Smoothelin Like 1	-2.93	1.24E-05
Pdlim3	PDZ And LIM Domain 3	-2.32	1.46E-05
Atp1a2	ATPase Na+/K+ Transporting Subunit Alpha 2	-2.83	1.48E-05
Synpo2l	Synaptopodin 2 Like	-2.65	2.08E-05
Sirpb1b	Signal-Regulatory Protein Beta 1B	2.75	3.35E-05
Lilra6	Leukocyte Immunoglobulin Like Receptor A6	2.25	3.83E-05
Trem1	Triggering Receptor Expressed on Myeloid Cells 1	2.56	3.85E-05
Sh3bgr	SH3 Domain Binding Glutamate Rich Protein	-2.44	6.19E-05
Trim54	Tripartite Motif Containing 54	-2.73	6.56E-05
Mogat2	Monoacylglycerol O-Acyltransferase 2	2.51	9.46E-05
Ryr1	Ryanodine Receptor 1	-2.55	1.46E-04
Map3k9	Mitogen-Activated Protein Kinase Kinase Kinase 9	2.17	1.47E-04
Des	Desmin	-2.58	1.54E-04
Hlf	HLF, PAR BZIP Transcription Factor	-2.38	1.62E-04
Mylpf	Myosin Light Chain, Phosphorylatable, Fast Skeletal Muscle	-2.56	1.70E-04
Stfa2l1	Stefin A2 Like 1	2.57	1.80E-04

Myoz3	Myozenin 3	-2.62	1.80E-04
Ttn	Titin	-2.34	2.21E-04
Pgam2	Phosphoglycerate Mutase 2	-2.61	2.27E-04
Cldn15	Claudin 15	2.24	2.65E-04
Kcna7	Potassium Voltage-Gated Channel Subfamily a Member 7	-2.39	4.32E-04
Ltb4r1	Leukotriene B4 Receptor 1	2.02	4.70E-04
Myo18b	Myosin XVIIIB	-2.16	4.94E-04
Dbp	D-Box Binding PAR BZIP Transcription Factor	-2.32	5.03E-04
Rasd2	RASD Family Member 2	-2.16	5.41E-04
AU023871	Megakaryocyte and Platelet Inhibitory Receptor G6b	2.48	5.75E-04
Apobec2	Apolipoprotein B MRNA Editing Enzyme Catalytic Subunit 2	-2.18	6.30E-04
Fcgr4	Fc Receptor, IgG, Low Affinity IV	2.04	7.17E-04
Myl1	Myosin Light Chain 1	-2.26	7.76E-04
Ankrd22	Ankyrin Repeat Domain 22	2.28	7.97E-04
Smyd1	SET and MYND Domain Containing 1	-2.34	8.27E-04
Sypl2	Synaptophysin Like 2	-2.10	8.27E-04
Ankrd1	Ankyrin Repeat Domain 1	-2.41	8.98E-04
Nlrp12	NLR Family Pyrin Domain Containing 12	2.19	1.19E-03
Ceacam10	Carcinoembryonic Antigen-Related Cell Adhesion Molecule 10	2.26	1.26E-03
Galnt9	Polypeptide N-Acetylgalactosaminyltransferase 9	2.00	1.41E-03
A530064D06Rik	RIKEN cDNA A530064D06 Gene	2.03	1.48E-03
Mgam	Maltase-Glucoamylase	2.25	1.57E-03
Nrap	Nebulin Related Anchoring Protein	-2.30	1.67E-03
Ppp1r3a	Protein Phosphatase 1 Regulatory Subunit 3A	-2.30	1.74E-03
Tigd4	Tigger Transposable Element Derived 4	-2.27	1.84E-03
Mpl	MPL Proto-Oncogene, Thrombopoietin Receptor	2.28	1.98E-03
Ano5	Anoctamin 5	-2.23	2.73E-03
Myom2	Myomesin 2	-2.19	2.95E-03
Tnfsf14	TNF Superfamily Member 14	2.12	3.43E-03
Xirp1	Xin Actin Binding Repeat Containing 1	-2.13	3.56E-03
Slc10a4	Solute Carrier Family 10 Member 4	-2.02	4.09E-03
Slco4c1	Solute Carrier Organic Anion Transporter Family Member 4C1	2.04	4.45E-03
Aqp9	Aquaporin 9	2.08	4.57E-03
Pvalb	Parvalbumin	-2.06	6.99E-03
Myoz1	Myozenin 1	-2.03	7.55E-03
Gp9	Glycoprotein IX Platelet	2.02	7.72E-03
2310002L09Rik	RIKEN cDNA 2310002L09 Gene	-2.03	8.03E-03

Supplementary Table 3: List of genes with corresponding Log2 fold change > absolute value of 2 and p-value < 0.01 from 17-week WT RNA sequencing analysis

Gene	Gene Description	Fold Change (Log2)	P Value
Pi15	Peptidase Inhibitor 15	4.34	1.07E-75
A2m	Alpha-2-Macroglobulin	2.77	4.69E-70
Greb1	Growth Regulation by Estrogen in Breast Cancer 1	5.57	7.06E-51
Lgi2	Leucine Rich Repeat LGI Family Member 2	5.42	9.32E-36
Trpc6	Transient Receptor Potential Cation Channel Subfamily Member 6	3.69	3.81E-33
Shank2	SH3 and Multiple Ankyrin Repeat Domains 2	3.39	8.02E-26
Gbp8	Guanylate Binding Protein 8	4.06	7.38E-25
Myo5c	Myosin VC	3.31	6.18E-20
Gbp4	Guanylate Binding Protein 4	2.84	9.54E-20
Mrvi1	Murine Retrovirus Integration Site 1 Homolog	2.41	1.38E-17
Gbp6	Guanylate Binding Protein 6	2.54	1.24E-15
Sftpa1	Surfactant Protein A1	4.06	5.18E-15
Mgl2	Macrophage Galactose N-Acetyl-Galactosamine Specific Lectin 2	3.18	1.05E-14
Micall2	MICAL Like 2	2.32	1.42E-11
Asb4	Ankyrin Repeat and SOCS Box Containing 4	3.35	1.68E-11
Hcn4	Hyperpolarization Activated Cyclic Nucleotide Gated Potassium Channel 4	3.57	2.49E-11
Ripk4	Receptor Interacting Serine/Threonine Kinase 4	3.27	3.24E-10
Fam196b	Family with Sequence Similarity 196 Member B	2.60	3.11E-06
Ear6	Eosinophil-Associated, Ribonuclease A Family, Member 6	-2.80	1.21E-05
Tnnc1	Troponin C1, Slow Skeletal And Cardiac Type	2.31	6.72E-05
Ntng1	Netrin G1	2.01	2.37E-04
Gdf3	Growth Differentiation Factor 3	2.19	6.71E-04
Zfp804a	Zinc Finger Protein 804A	2.11	4.35E-03
Ear2	Eosinophil-Associated, Ribonuclease A Family, Member 2	-2.04	4.72E-03
Gbp10	Guanylate Binding Protein 10	2.05	6.58E-03
Ctse	Cathepsin E	-2.02	7.89E-03

Supplementary Table 4: List of genes with corresponding Log2 fold change > absolute value of 2 and p-value < 0.01 from 17-week ERαKO RNA sequencing analysis

Gene	Gene Description	Fold Change (Log2)	P Value
Rbm24	RNA Binding Motif Protein 24	-3.65	1.17E-10
Scn4a	Sodium Voltage-Gated Channel Alpha Subunit 4	-2.55	3.22E-04

Supplemental Table 5: List of genes significantly regulated in both 7-week and 17-week WT mandibular condylar fibrocartilage at Log2 fold change > absolute value of 2 and p-value < 0.01 from RNA sequencing analysis

Gene	7-week Fold Change (Log2)	7-week P Value	17-week Fold Change (Log2)	17-week P Value
Trpc6	3.91	7.87E-103	3.69	3.81E-33
Pi15	3.56	1.38E-46	4.34	1.07E-75
Lgi2	4.77	1.02E-32	5.42	9.32E-36
A2m	2.62	6.03E-32	2.77	4.69E-70
Greb1	5.20	1.22E-28	5.57	7.06E-51
Sftpa1	5.69	2.70E-26	4.06	5.18E-15
Asb4	4.81	3.15E-25	3.35	1.68E-11
Shank2	3.75	8.71E-19	3.39	8.02E-26
Myo5c	3.62	1.04E-16	3.31	6.18E-20
Gbp4	2.08	5.32E-14	2.84	9.54E-20
Gbp8	3.05	9.21E-13	4.06	7.38E-25
Mrvi1	2.11	3.88E-12	2.41	1.38E-17
Gbp6	2.71	9.33E-12	2.54	1.24E-15
Mgl2	2.35	2.80E-11	3.18	1.05E-14
Ear6	-3.39	1.05E-05	-2.80	1.21E-05
Fam196b	3.25	1.69E-05	2.60	3.11E-06
Hcn4	2.60	6.54E-04	3.57	2.49E-11
Ripk4	2.68	1.17E-03	3.27	3.24E-10
Ctse	-2.69	1.52E-03	-2.02	7.89E-03

Supplementary Table 6: Effect of estradiol treatment on ERα (ESR1) gene expression in RNA sequencing analysis of the mandibular condylar fibrocartilage from both 7-week and 17-week old WT female mice

Gene	Gene Description	Fold Change (Log2)	P Value
Esr1 (7-week)	Estrogen receptor 1	-0.49	0.67
Esr1 (17-week)	Estrogen receptor 1	-0.58	0.24

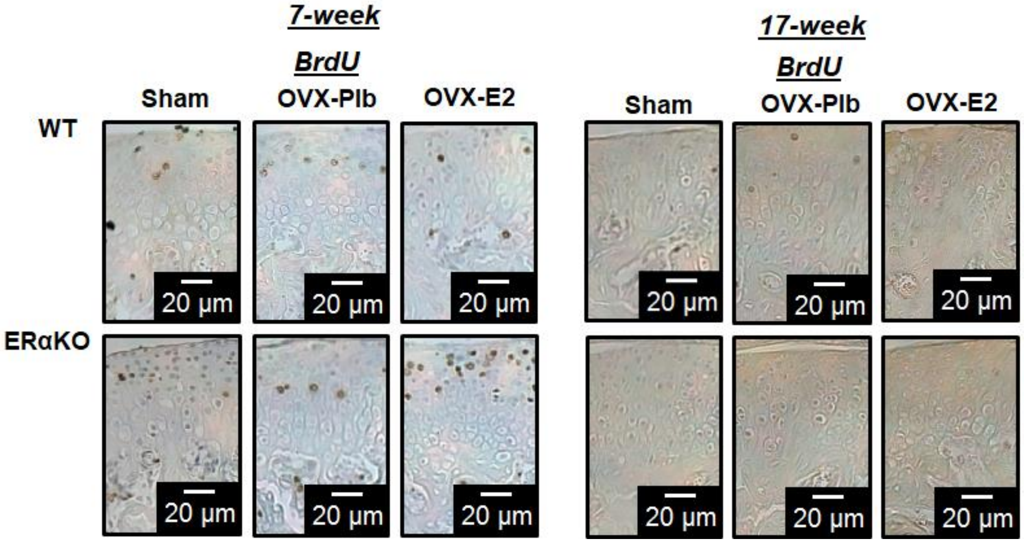

Supplementary Figure 1: Higher magnification images of BrdU staining in both 7- and 17-week samples for all groups

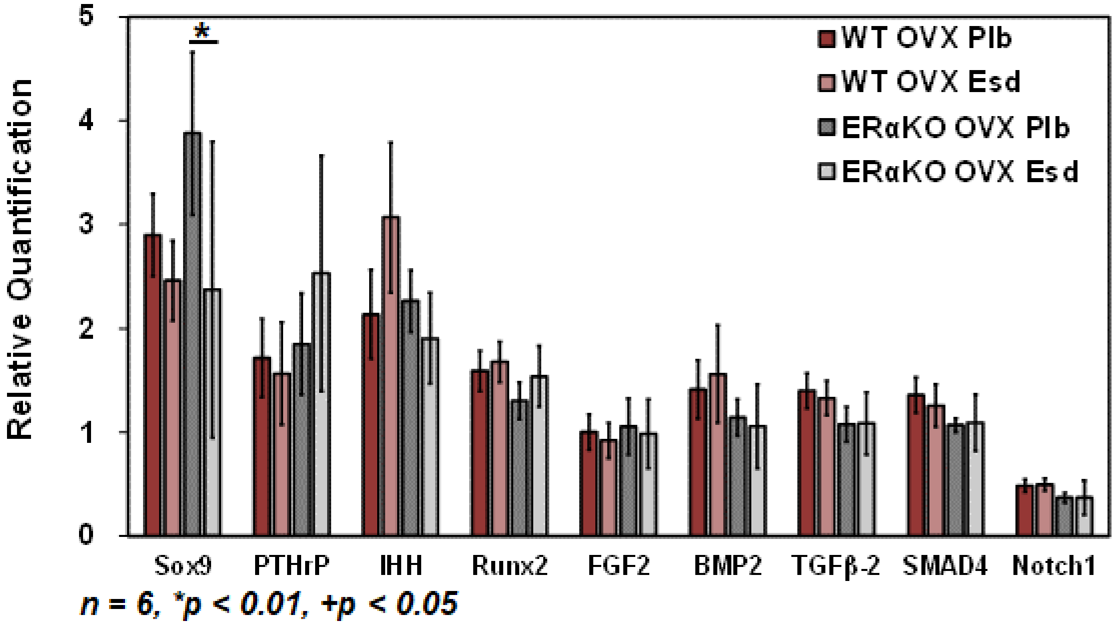

Supplementary Figure 2: Mandibular condylar fibrocartilage-related genes in 7-week old female mice. Gene expression of Sox9, Pthrp, Ihh, Runx2, FGF2, BMP2, TGFβ-2, SMAD4, and Notch1 from 7-week old mice. All values represent means ± standard deviation. n = 6 for all data, ^p < 0.01, *p<0.05.

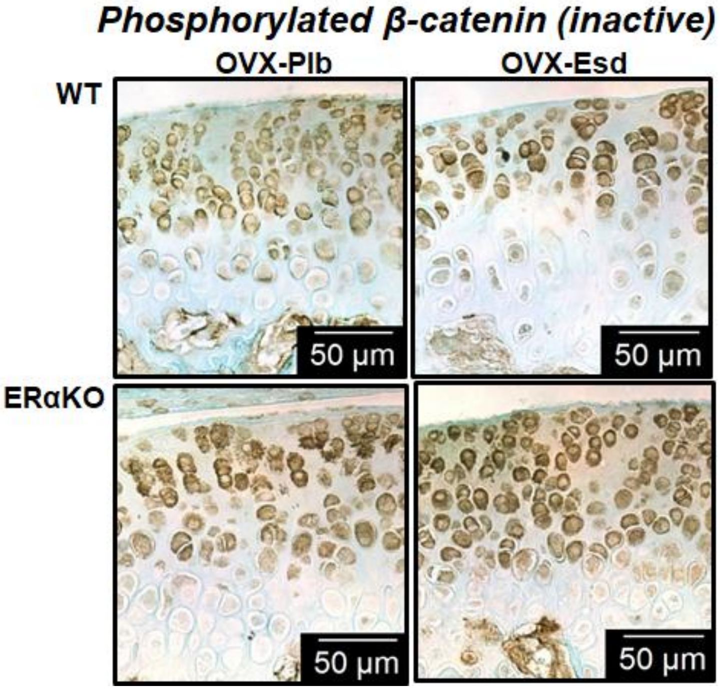

Supplementary Figure 3: Immunohistochemical staining of inactive β-catenin in 7-week old WT and ERαKO samples Representative phosphorylated β-catenin immunohistochemical images in WT and ERαKO female mice with placebo and estradiol treatment.

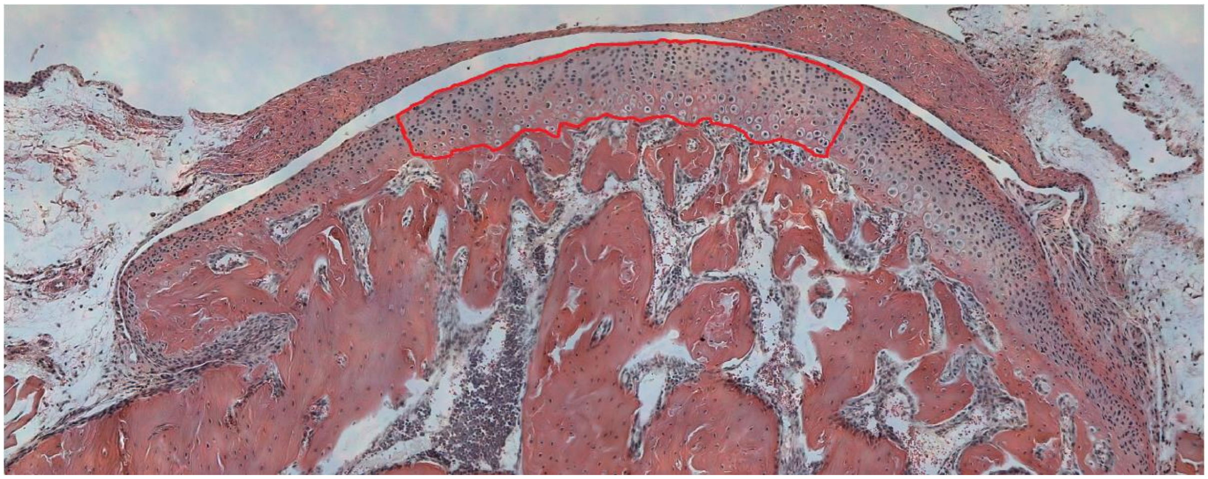

Supplementary Figure 4: Delineation of region utilized to quantify fibrocartilage thickness in mandibular condylar H&E sections

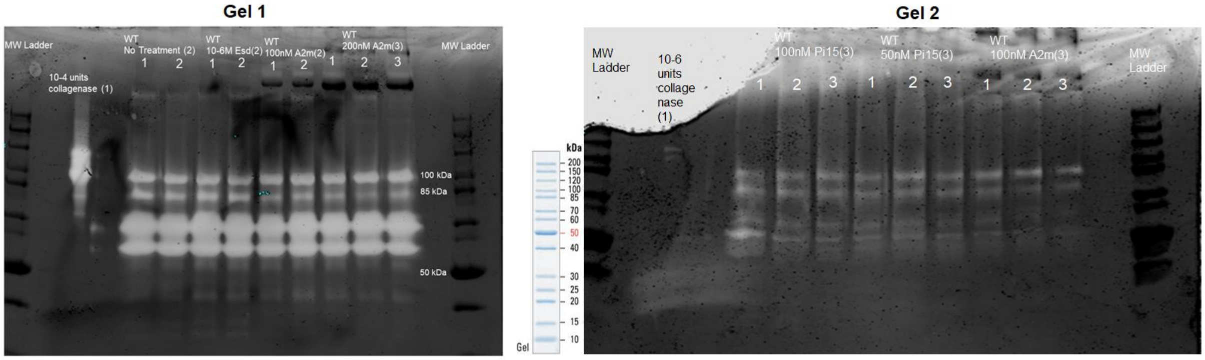

Supplementary Figure 5. Full length zymography gels using 10% gelatin substrates with supernatant analyzed after organ culture with A2m and Pi15 treatment using collagenase as a positive control.
